# A bird’s-eye view of the biological mechanism and machine learning prediction approaches for cell-penetrating peptides

**DOI:** 10.3389/frai.2024.1497307

**Published:** 2025-01-07

**Authors:** Maduravani Ramasundaram, Honglae Sohn, Thirumurthy Madhavan

**Affiliations:** ^1^Department of Genetic Engineering, Computational Biology Lab, School of Bioengineering, SRM Institute of Science and Technology, SRM Nagar, Chennai, India; ^2^Department of Chemistry and Department of Carbon Materials, Chosun University, Gwangju, Republic of Korea

**Keywords:** cell-penetrating peptides, mechanism, machine learning, random forest, support vector machine, artificial neural network

## Abstract

Cell-penetrating peptides (CPPs) are highly effective at passing through eukaryotic membranes with various cargo molecules, like drugs, proteins, nucleic acids, and nanoparticles, without causing significant harm. Creating drug delivery systems with CPP is associated with cancer, genetic disorders, and diabetes due to their unique chemical properties. Wet lab experiments in drug discovery methodologies are time-consuming and expensive. Machine learning (ML) techniques can enhance and accelerate the drug discovery process with accurate and intricate data quality. ML classifiers, such as support vector machine (SVM), random forest (RF), gradient-boosted decision trees (GBDT), and different types of artificial neural networks (ANN), are commonly used for CPP prediction with cross-validation performance evaluation. Functional CPP prediction is improved by using these ML strategies by using CPP datasets produced by high-throughput sequencing and computational methods. This review focuses on several ML-based CPP prediction tools. We discussed the CPP mechanism to understand the basic functioning of CPPs through cells. A comparative analysis of diverse CPP prediction methods was conducted based on their algorithms, dataset size, feature encoding, software utilities, assessment metrics, and prediction scores. The performance of the CPP prediction was evaluated based on accuracy, sensitivity, specificity, and Matthews correlation coefficient (MCC) on independent datasets. In conclusion, this review will encourage the use of ML algorithms for finding effective CPPs, which will have a positive impact on future research on drug delivery and therapeutics.

## 1 Introduction

Peptide prediction is critical for the recognition of novel and systematic peptide-based therapeutics (Gautam et al., 2013). Two major hindrances to the development of drugs are poor delivery and truncated bioavailability of drug molecules in therapy (Manavalan et al., 2018). The plasma membrane is particularly permeable and endures as a prime barrier for many therapeutic cargos. Several delivery systems have been evolved to outlive this barrier (Gao et al., 2007). Available delivery techniques can lead to high toxicity, immunogenicity, and insufficient delivery yield. CPPs have accomplished much appreciation as an outstanding delivery module since they have high bioavailability (Heitz et al., 2009). CPPs, also called “Trojan” peptides, and protein transduction domains (PTDs) are roughly around the length of 5 to 30 amino acids that can enter cell membranes via energy-dependent and independent mechanisms. CPPs have a remarkable ability to obliquely eukaryotic membranes without deteriorating the outer membrane (Gautam et al., 2013). CPPs can carry diverse particles, such as peptides, proteins, drugs, nucleic acids, siRNAs, and nanoparticles, across the lipid bilayer (Hansen et al., 2008; Bechara and Sagan, 2013; Brasseur and Divita, 2010; Sanders et al., 2011). With its high specificity, affinity, low toxicity, and relatively low cost, CPP enables therapeutic medications to overcome the limitations of small molecules (Guerrero-Vázquez et al., 2023). Almost every aggregate/drug molecule can be carried into the cell once coupled to CPP (Fonseca et al., 2009). Therefore, CPPs have terrific therapeutic potential, particularly in the area of drug delivery. They have become a hotspot for gene and anti-tumor drug research (Kamei et al., 2016; Pang et al., 2015).

Using peptides as drugs is limited by their low stability, limited membrane penetration, reduced solubility, quick clearance, limited oral bioavailability, and elevated production costs (Craik et al., 2013; Marqus et al., 2017; Lee et al., 2019). The identification of appropriate medicinal peptides involves biological wet lab methods and computational-assisted identification methods. The *in vitro* method is an expensive, challenging, and time-consuming procedure. To control these limitations, new and effective computational approaches have been developed by researchers (Li et al., 2020). Such ideas could be used to screen peptides before their synthesis, thereby accelerating peptide discovery (Manavalan et al., 2017). ML-based computational ideologies can serve as swift and inexpensive pre-screening tools to proficiently cover the diverse and crucial sequence margin, thereby facilitating and this will rationalize the process of peptide discovery (Tang et al., 2016; Sidey-Gibbons and Sidey-Gibbons, 2019; Li et al., 2019; Basith et al., 2020). ML techniques have been put forth to discover novel CPPs that may be further investigated experimentally (Guerrero-Vázquez et al., 2023).

Classification of CPPs computationally from peptide sequences was pitched in 2005 (Hällbrink et al., 2005). Followed by this, various ML-based CPP predictors have been developed, which include artificial neural networks (ANN) (Dobchev et al., 2010; Holton et al., 2013; Cai et al., 2021; de Oliveira et al., 2021; Manavalan and Patra, 2022; Park et al., 2023; Zhang et al., 2023), support vector machine (SVM) (Gautam et al., 2013; Sanders et al., 2011; Tang et al., 2016; de Oliveira et al., 2021; Manavalan and Patra, 2022; Zhang et al., 2023; Fu et al., 2020; Fu et al., 2019), extremely randomized tree (ERT) (Manavalan et al., 2018; Manavalan and Patra, 2022), gradient-boosted decision trees (GBDT) (Arif et al., 2020), light gradient boosting machine (LGBM) (Maroni et al., 2024), kernel extremely learning machine (KELM) (Pandey et al., 2018), and random forest (RF) (de Oliveira et al., 2021; Manavalan and Patra, 2022; Chen et al., 2015; Diener et al., 2016; Wei et al., 2017b; Wei et al., 2017a; Kumar et al., 2018; Qiang et al., 2020; Wei L. et al., 2019). In the past, around 15 CPP predictors have been reviewed and compared based on datasets and prediction strategies (Guerrero-Vázquez et al., 2023; Wei H. H. et al., 2019). A meticulous comparative analysis of cutting-edge ML techniques in the investigation of CPPs is crucial given the increasing interest in their applications in drug delivery, molecular treatment, and biomedicine. In this review, the CPPs penetrating mechanism through cell membranes was highlighted. A comparison of contemporary prediction methods for CPP was conducted based on their accuracy and MCC on training and independent datasets. We have included 26 prediction methods that were exclusively constructed for forecasting CPPs. Among 26, 5 predictors followed a 2-layer framework (Wei et al., 2017b; Manavalan et al., 2018; Fu et al., 2020; Arif et al., 2021; Manavalan and Patra, 2022). Every approach that has been examined fits within the framework of supervised learning, and all of the positive samples in the datasets were empirically curated CPPs (Agrawal et al., 2016). All of these methods were thoroughly investigated to identify the statistical indices, advantages, and pitfalls. Balanced and imbalanced datasets were scrutinized concerning each prediction method. We anticipate that this review will help biologists with the appropriate computational tools for CPP-dependent therapeutics.

## 2 Overview of the mechanism of CPP internalization

**Figure 1:**
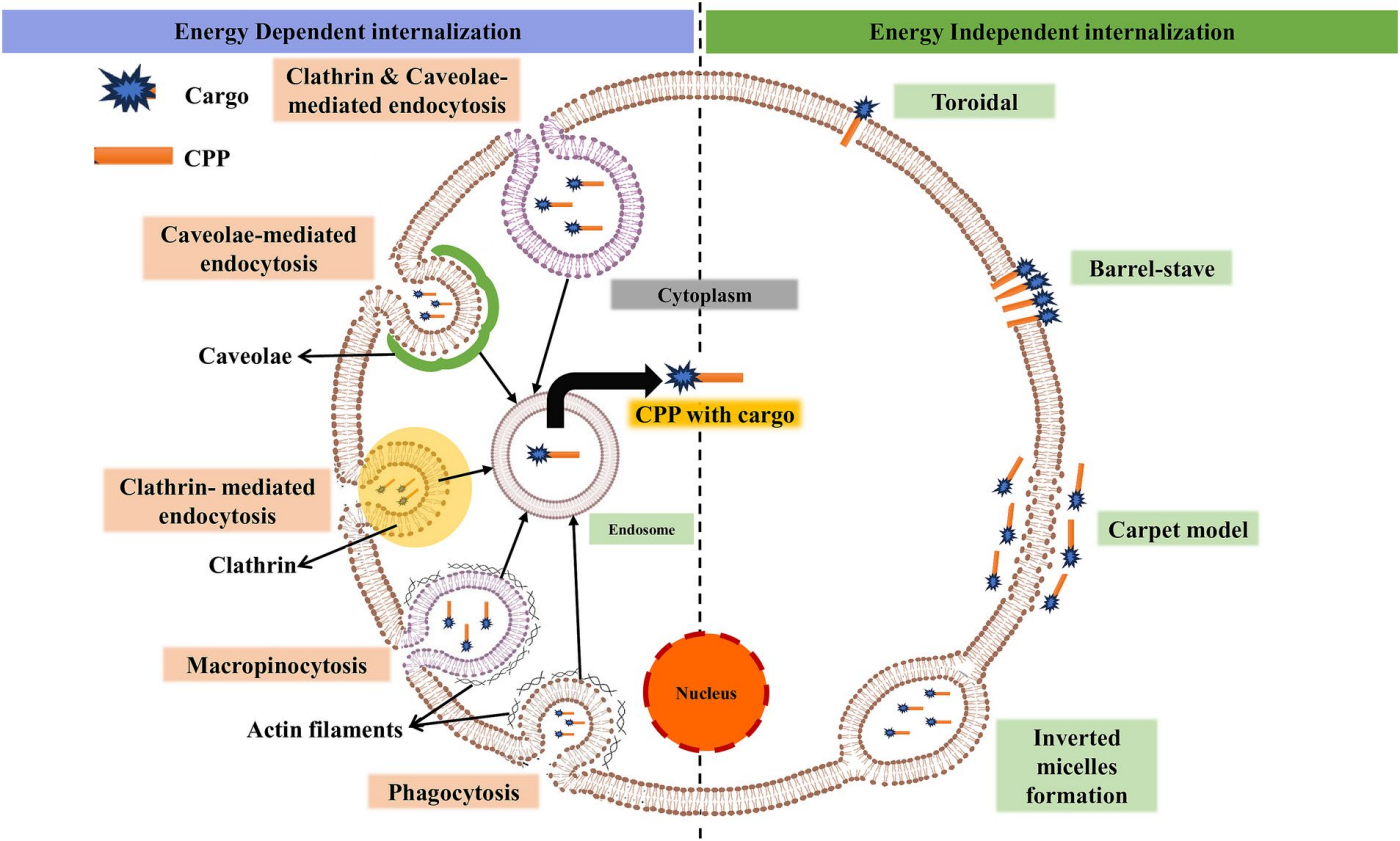
Illustration of the basic mechanism of cell-penetrating peptides for intracellular invagination into cells. Cargo can be a drug, protein, micromolecule, siRNA, etc. (created with BioRender.com/k22o427).

Although numerous studies have been conducted on CPPs, the mechanism by which they enter the cell remains unclear and controversial in some cases (Bechara and Sagan, 2013). The mechanism of CPP uptake into cells is crucial for optimizing the efficiency and safety of intracellular delivery, which may be suitable for a specific cargo (Madani et al., 2011). The use of ML models for accurate prediction and design relies on significant input features such as sequence motifs, hydrophobicity, charge, peptide length, and the secondary structure of CPPs. Understanding the biological mechanism of CPPs is crucial for researchers to design challenging features that incorporate the fundamental aspects of CPP functioning, such as peptide-lipid interactions (Copolovici et al., 2014). In the absence of biological understanding, feature selection for ML prediction might miss critical aspects, resulting in poor model performance (Yadahalli and Verma, 2020). The cellular absorption pathways for CPPs include an energy-independent pathway and an endocytic pathway, each with unique characteristics (Ruseska and Zimmer, 2020; Gori et al., 2023). Figure 1 shows different methods of the intracellular mechanism of CPP penetration into cells.

In the presence of endocytic inhibitors and at low temperatures, either energy-independent or direct penetration occurs (Trabulo et al., 2010). The negatively charged phospholipid bilayer membrane and the peptide often interact electrostatically during direct penetration (Wallbrecher et al., 2017). Following contact, there is either a transient or persistent membrane instability, which allows the peptide to enter the cytosol. Direct penetration is a one-step process that involves the development of pores, carpet models, and inverted micelles, among other processes (Ruczynski et al., 2014). The inverted micelles are formed due to membrane invagination, which traps the CPP with conjugated cargo at low concentrations (Derossi et al., 1998). Penetratin was reported to be the first CPP to follow an inverted micelle mechanism (Alves et al., 2010). Pep-1 and MPG are amphiphilic CPPs that undergo pore formation through direct penetration (Heitz et al., 2009; Deshayes et al., 2006). The pore formation mechanism involves two different models: the barrel-stove and the toroidal. Bundle formation through peptide interaction with the plasma membrane happens in the barrel-stove model (Bechara and Sagan, 2013). The inward bending of the lipid monolayer leads to hydrophilic pores in the toroidal model. Carpet-like association of the peptide itself on the lipid bilayer was found in the carpet-like model (Pouny et al., 1992). Interaction happens between the hydrophobic regions of the peptide and lipid bilayer, allowing the peptide to penetrate inside the cells due to changes in membrane conformation (Galanth et al., 2009).

Due to energy expenditure, macromolecular internalization occurs through endocytosis (energy-dependent) (Zhao et al., 2011). This process occurs through two types: phagocytosis and pinocytosis. The uptake of specialized cells (cell eating), like monocytes and neutrophils, is phagocytosis (Desale et al., 2021). Liquids and solutes uptake (cell drinking) is pinocytosis. Pinocytosis involves four mechanisms (Conner and Schmid, 2003). (i) Macropinocytosis is the formation of membrane protrusions due to polymerization of actin (Wadia et al., 2004; Futaki et al., 2007). Kinases and GTPases are the key enzymes involved in this process (Lim and Gleeson, 2011). (ii) Clathrin-mediated endocytosis (CME) is a process for nutrient uptake in all mammalian cells (Haucke and Kozlov, 2018). It is a receptor-guided process where vesicles covered by clathrin are converted into endosomes and released into the cytosol (Veldhoen et al., 2006; Arukuusk et al., 2013). (iii) Caveolae-mediated endocytosis (CVME) is the formation of caveolae, which are cave-like invaginations on the inner side of the cell under the guidance of caveolins and cavins (Kovtun et al., 2015). Glypicans with TAT, azurin, and chicken anemia virus (CVP1) are peptides that undergo internalization through this pathway (Nabi and Le, 2003; De Pasquale and Pavone, 2020; Hao et al., 2022; Taylor et al., 2009). (iv) Clathrin- and caveolae-independent endocytosis undergo specific uptake of glycolipids, raft-associated receptors, cholesterol, and GPI-anchored proteins (Damm et al., 2005; Johannes and Lamaze, 2002; Parton and Richards, 2003). Protamine CPP for siRNA delivery is the best example of independent endocytosis.

## 3 General machine learning scheme for cell-penetrating peptide prediction

A thorough examination of different methods revealed that the machine learning approach for the prediction of CPP involved four steps: the first step involved the construction of reliable datasets, namely, training and independent datasets. CPPsite 2.0 is the most abundant and largest database for experimentally validated CPPs available at http://crdd.osdd.net/raghava/cppsite/ (Agrawal et al., 2016). The majority of the predictors have retrieved datasets from this database. The CD-HIT (Cluster Database at High Identity with Tolerance) program was used to remove sequence redundancy to prevent overfitting of the model (Fu et al., 2020; Diener et al., 2016; Qiang et al., 2020). The second step was the extraction of optimal feature descriptors to train the classifier. The third step involved model training and evaluation of training and independent datasets based on feature encodings. K*-*fold cross-validation has been widely used for the evaluation of the algorithm’s performance. The evaluation indices included to assess the prediction performance were accuracy (ACC), sensitivity (SN), specificity (SP), and Matthews correlation coefficient (MCC) (Park et al., 2023). All the existing prediction methods follow these statistical indicators. The values were enumerated as follows:


$$SN=\left(\frac{TP}{TP+FN}\;\right)\times 100\%$$

$$SP=\left(\frac{TN}{TN+FP}\right)\times 100\%$$

$$ACC=\left(\frac{TP+TN}{TP+TN+FP+FN}\right)\times 100\%$$

$$MCC=\frac{TP\times TN-FP\times FN}{\sqrt{\left(TP+FN\right)\left(TP+FP\right)\left(TN+FN\right)\left(TN+FP\right)}}$$

TP indicates the number of true +ve samples (CPPs); TN depicts the number of true −ve samples (non-CPPs); FP represents the number of false +ve samples, non-CPPs speculated to be CPPs; FN represents the number of false −ve samples, CPPs predicted to be non-CPPs (Park et al., 2023). The area under the curve (AUC) from the receiver operating characteristic curve (ROC) was used to visually represent the model’s interpretation (Manavalan and Patra, 2022; Kumar et al., 2018). A web server was developed for certain predicted models that demonstrated robust performance (Gautam et al., 2013; Holton et al., 2013; Diener et al., 2016; Tang et al., 2016; Wei et al., 2017b; Wei et al., 2017a; Kumar et al., 2018; Manavalan et al., 2018; Pandey et al., 2018; Qiang et al., 2020; Wei L. et al., 2019; de Oliveira et al., 2021; Manavalan and Patra, 2022). All the information related to the predictors is available on the web server, which will be useful for the researchers in providing insights for further research to develop advanced predictions (Basith et al., 2020).

## 4 State-of-the-art methods

In our article, we identified 26 predictors of cell-penetrating peptides that have been reported to date. Table 1 highlights information on the CPP predictors evaluated in this review. The predictor’s name, number of datasets, feature encodings, classifier used, evaluation indices, accuracy of prediction, and web server information were mentioned to provide insights about these methods.

**Table 1:** List of currently available CPP predictors evaluated in this review.

Predictor/author’s name	Classifier	Year	Feature encodings	Dataset size (positive/negative)		Assessment strategy (CV)	Accuracy (%)		Web server availability	References
				Training dataset	Independent dataset		Training	Independent		
Dobchev et al.	MLP	2010	PCA	49/10	23/2	3-Fold	83.1	92.0	NA	Dobchev et al. (2010)
Sanders et al.	SVM	2011	PCP	111/34	-	10-Fold	91.7	-	NA	Sanders et al. (2011)
CellPPD	SVM	2013	BPP	708/708	99/99	5-Fold	97.4	81.3	http://crdd.osdd.net/raghava/cellppd/	Gautam et al. (2013)
CPPpred	N-to-1 NN	2013	Motif	74/100	47/47	5-Fold	77.6	82.9	http://bioware.ucd.ie/cpppred	Holton et al. (2013)
Chen et al.	RF	2015	PseAAC	111/34	-	10-Fold	83.4	-	NA	Chen et al. (2015)
DCF	RF	2016	PCPs	1,267/1,267	-	4-Fold	90.0	-	http://bis.ifc.unam.mx/en/software/dcf	Diener et al. (2016)
C2Pred	SVM	2016	DPC	411/411	-	5-Fold	83.6	-	http://lin-group.cn/server/C2Pred	Tang et al. (2016)
SkipCPP-Pred	RF	2017	Adaptive k-skip-2-gram	462/462	-	LOOCV	90.6	-	http://server.malab.cn/SkipCPP-Pred/Index.html	Wei et al. (2017a)
CPPred-RF	RF	2017	PC-PseAAC, SC-PseAAC, ASDC, PCP	462/462	-	LOOCV	91.6	-	http://server.malab.cn/CPPred-RF	Wei et al. (2017b)
CellPPD-Mod	RF	2018	2D, 3D, Fingerprint descriptors	582/582	150/150	5-Fold	95.1	92.3	http://webs.iiitd.edu.in/raghava/cellppdmod	Kumar et al. (2018)
MLCPP	ERT	2018	AAC and PCP	427/427	311/311	10-Fold	88.3	89.6	www.thegleelab.org/MLCPP	Manavalan et al. (2018)
KELM-CPPpred	KELM	2018	AAC, PseAAC, DPC, hybrid motifs	408/408	96/96	10-Fold	86.2	83.1	http://sairam.People.iitgn.ac.in/KELM-CPPpred.html	Pandey et al. (2018)
PEPred-suite	RF	2019	10 feature encodings	370/370	92/92	10-Fold	91.2	NR	http://server.Malab.cn/PEPred-Suite	Wei L. et al. (2019)
Fu et al.	SVM	2019	GAAC, CKSAAGP, GDPC, CTD	462/462	96/96	LOOCV	92.3	84.4	NA	Fu et al. (2019)
G-DipC	XGB	2020	DPC	1,223/ 1,223	-	5-Fold	83.9	-	NA	Wang et al. (2019)
CPPred-FL	RF	2020	9 feature encodings	462/462	-	10-Fold	92.1	-	http://server.Malab.cn/CPPred-FL	Qiang et al. (2020)
StackCPPred	SVM	2020	PseRECM	462/462	-	10-Fold	94.5	-	NA	Fu et al. (2020)
TargetCPP	GBDT	2020	CPSR, CTD, SAAC, ITF	462/462	111/34	LOOCV	93.5	88.4	NA	Arif et al. (2020)
BChemRF-CPPred	ANN, SVM, GPC	2021	AAC, PseAAC, DPC	300/300	75/75	10-Fold	87.6	90.6	http://comptools.linc.ufpa.br/BChemRF-CPPred	de Oliveira et al. (2021)
ITP-Pred	CNN-BiLSTM	2021	AAC, PCP	370/370	92/92	5-Fold	89.0	95.1	NA	Cai et al. (2021)
DeepCPPred	CDF	2022	PSSM, RECM, SMR, RSIV	462/462	-	5-Fold	93.0	-	NA	Arif et al. (2021)
MLCPP 2.0	7 ML classifiers	2022	17 feature encodings	573/573	157/2184	10-Fold	91.3	93.4	https://balalab-skku.org/mlcpp2/	Manavalan and Patra (2022)
SiameseCPP	SNN	2023	CL features	462/462	-	NR	96.1	-	NA	Zhang et al. (2023)
AiCPP	LSTM	2023	9-mer approach	1,249/1,097	150/150	10-Fold	NR	86.0	NA	Park et al. (2023)
PractiCPP	PractiCPP	2024	SF, LSF, PTF	462/462	649/649,000	10-Fold	95.6	80.5	NA	Shi et al. (2024)
LightCPPgen	LGBM	2024	375 features	573/573	157/2184	10-Fold	NR	96.2	NA	Maroni et al. (2024)

NR, not reported; NA, not available; MLP, multilayer perceptron; ANN, artificial neural network; SVM, support vector machine; RF, random forest; ERT, extremely randomized tree; KELM, kernel extremely learning machine; XGB, extreme gradient boosting; GBDT, gradient-boosted decision trees; GPC, Gaussian process classification; CNN-BiLSTM, convolutional neural network-bidirectional long short-term memory; SNN, siamese neural network; LSTM, long-short term memory; LGBM, light gradient boosting machine; QSAR, quantitative structure-active relationship; PCA, principal component analysis; PCP, physicochemical properties; AAC, amino acid composition; BPP, binary profiles of pattern; DPC, dipeptide composition; GAAC, grouped amino acid composition; CKSAAGP, composition of k-spaced amino acid group pairs; GDPC, grouped dipeptide composition; PseAAC, pseudo amino acid composition; PC-PseAAC, parallel correlation-pseudo amino acid composition; SC-PseAAC, series correlation-pseudo amino acid composition; PseRECM, pseudo residue pairwise energy content matrix; ASDC, adaptive skip dipeptide composition; SAAC, split-amino acid composition; ITF, information theory features; CPSR, composition protein sequence representation; CTD, composition-transition-distribution; CDF, cascade deep forest; PSSM, position-specific scoring matrix; RECM, residue energy contact matrix; SMR, substitution matrix representation; RSIV, reduced sequence and index-vectors; CL, contrastive learning; SF, sequential features; LSF, local structure features; PTF, pretrained features; LOOCV, leave-one-by-one cross validation; CV, cross validation.

The model with QSAR features as input to MLP (multilayer perceptron), which is a type of ANN, was thrived by Dobchev. He performed a PCA with STASTICA for the MLP attributes with 101 peptide datasets. The false +ve and false -ve samples were prohibited during model progression and achieved 83% accuracy over the training set and ~ 100% accuracy over the validation set (Dobchev et al., 2010). Sanders evolved an SVM classifier with 61 different features, based on PCP. Features were screened using a wrapper-based selection. A 10-fold cross-validation was used on 111 benchmarks and 34 test datasets to achieve accuracy (ACC), sensitivity (SN), and specificity (SP) of 91.72, 91.70, and 12.70%, respectively. Fluorescence microscopy was then used to empirically verify the cell penetration functionality, and a quantitative uptake analysis of the peptides was carried out (Sanders et al., 2011).

CellPPD is an SVM-based predictor that utilizes AAC, DPC, BPP, and PCP as input features. The training and independent datasets were retrieved from the CPPsite (Agrawal et al., 2016; Gautam et al., 2012). The SVM-BPP model achieved an accuracy of 81.30% on independent datasets. Training datasets with hybrid features (BPP-based motif) achieved the highest performance with SN, SP, and ACC at 98.15, 96.58, and 97.40%, respectively. This is the first web server that was easy to use. It provided the opportunity to create analog devices with improved cell penetration capabilities (Gautam et al., 2013).

A web server CPPpred with an N-to-1 neural network using motif-based features as input progressed with 174 training and 94 independent datasets. These datasets were generated by performing redundancy removal using BLAST. A 5-fold CV was performed that achieved moderate performance with 77.60 and 82.98% accuracy for training and independent datasets, respectively (Holton et al., 2013). An RF model with pseudo-amino acid composition as a feature input was developed. Curated CPPs and non-CPPs were retrieved from Sanders’s method (Sanders et al., 2011). For the representation of each sample, 270 features were employed. The max-relevance and min-redundancy (mRMR) encoding method was performed to understand the importance of optimal features for model building. The incremental feature selection (IFS) method and the random forest were used to construct an optimal prediction method and extract the best combination of features. In comparison, the PseAAC-RF method achieved 83.40% accuracy on training datasets evaluated using the 10-fold CV technique (Chen et al., 2015).

The DCF tool was developed to design multifunctional CPPs using 27 different PCP features. Random forest algorithm predicted multifunctional CPPs with 90% accuracy evaluated using 4-fold CV on training datasets (Diener et al., 2016). An SVM-based C2Pred tool was developed with dipeptide composition as a feature descriptor on benchmark datasets of 411 CPPs and 411 non-CPPs retrieved from CPPsite2.0 (Agrawal et al., 2016). The CD-HIT program was employed for redundancy removal. This tool was developed to achieve better accuracy than the aforementioned methods. The SN, SP, and ACC were achieved at 81.50, 85.60, and 83.60%, respectively (Tang et al., 2016).

SkipCPP-Pred was developed using an RF classifier with a k-skip-2 g feature algorithm that achieved ACC, SN, and SP of 90.60, 88.50, and 92.60% on training datasets with LOOCV (leave-one-out cross-validation), which is a validation technique to estimate the reliability of achieved statistical results. Rapid CPP prediction was accomplished by the utilization of sequential information (Wei et al., 2017a). CPPred-RF is the first tool that simultaneously predicts both CPPs and their uptake efficiency. The RF algorithm was executed using four sequence-based descriptors, namely, PC-PseAAC, SC-PseAAC, ASDC, and PCP. mRMR and sequential forward search (SFS) were employed for prioritizing the essential features and traversing through the subset of effective features, respectively. Benchmark datasets achieved better performance using the LOOCV strategy using ACC, SN, and SP of 91.60, 90.50, and 92.60%, respectively (Wei et al., 2017b).

CellPPDMod was developed to predict and evaluate modified CPPs. This study was performed using different combinations of descriptors, such as seventeen 2D, six 3D, and twenty-seven fingerprints, which achieved robust performance in the RF-based model. Feature selection was performed using the “CfsSubsetEval” evaluator to obtain these features. The training datasets used for internal validation achieved ACC, SN, and SP values of 95.10, 95.19, and 95.02%. The independent datasets achieved ACC, SN, and SP of 92.33, 91.3, and 93.3% with a 5-fold CV evaluation (Kumar et al., 2018).

The MLCPP is a two-layer prediction model that has been developed to predict CPPs/non-CPPs in the first layer and their effectiveness of uptake in the second layer. This method involved five different feature compositions, namely, AAC, DPC, AAI, CTD, and PCP, employed with four different ML methods such as SVM, ERT, RF, and *k-*nearest neighbor (*k-*NN). MLCPP was the first to employ an ERT-based model that achieved robust performance with hybrid features (AAC and PCP combination) on both training and independent datasets. On training datasets, the first layer prediction achieved ACC, SN, and SP of 88.30, 91.90, and 84.50%, respectively. The independent datasets achieved ACC, SN, and SP of 89.60, 93.30, and 85.80%, respectively, with a 10-fold CV technique. An easy-to-use web server has been proposed for encouraging further prediction by researchers (Manavalan et al., 2018).

KELM-CPPpred is an advanced tool for the prediction of CPPs developed exclusively with hybrid features, which involved 3 combinations—AAC, DPC, and PseAAC. The kernel extreme learning machine (KELM) model outperformed existing predictions, such as ANN, SVM, and RF. A 10-fold CV was used for the evaluation of the performance. Training datasets achieved ACC, SN, and SP of 86.21, 82.61, and 89.56%, respectively. Independent datasets achieved scores for ACC, SN, and SP of 83.10, 78.72, and 88.05%, respectively. A user-friendly server was built for promoting further research (Pandey et al., 2018).

A bioinformatics tool with an adaptive feature representation called PEPred-Suite was developed with an RF algorithm. Various sequence-based descriptors were used to develop different RF-based models. The benchmark datasets followed a 10-fold assessment technique and performed better with ACC, SN, and SP of 91.20, 90.30, and 92.20%, respectively. The independent datasets achieved 95.20% accuracy, thus indicating the robust performance of the developed model (Wei L. et al., 2019). An SVM algorithm incorporating RFE (recursive feature elimination) and CBR (correlation bias reduction) was implemented on CPP benchmark datasets from CPP924 with four different feature encodings. The algorithm achieved outstanding prediction with the CTDC feature technique and the jackknife test strategy. The ACC, SN, and SP of the training datasets were 92.3, 91.8, and 92.9%, respectively. ACC, SN, and SP of the independent datasets were 84.4, 82.3, and 86.5%, respectively (Fu et al., 2019).

G-DipC is a method of improved feature representation that was developed using the XGBoost algorithm for shorter sequences. Numerous training datasets were evaluated using a 5-fold CV strategy. To minimize the cost of computation, linear discrimination analysis (LDA) was utilized. This method performed better with dipeptide composition, with ACC, SN, and SP of 83.98, 65.28, and 70.67%, respectively (Wang et al., 2019). CPPred-FL prospered in predicting large-scale identification of CPPs. Nine different feature encodings, such as CTD, AAC, PC-PseAAC, SC-PseAAC, GGAP DPC, ASDC, OLP (overlapping property features), BIT20 (binary profile algorithm), BIT21 (position-specific algorithm with PCP), and the N + C terminal approach, are utilized to determine CPPs in RF classifiers. A 10-fold validation strategy was implemented, which achieved better performance with ACC, SN, and SP of 92.10, 92.40, and 91.80% (Qiang et al., 2020).

StackCPPred used a 2-layer strategic approach for CPP prediction that employed the training datasets from CPPred-RF (Wei et al., 2017b). Of the three different feature encodings implemented with the SVM classifier, PseRECM (pseudo residue pairwise energy content matrix) achieved better performance in model prediction when evaluated using a ten-fold evaluation strategy. The ACC, SN, and SP of the predicted SVM-PseRECM model were 94.50, 94.20, and 94.80% (Fu et al., 2020). TargetCPP was a model built using a gradient-boosted decision trees (GBDT) and four different feature algorithms. The mRMR feature selection method was used to categorize optimal feature subsets. The leave-one-out CV technique was utilized to analyze the performance of training and independent datasets. Training datasets achieved ACC, SN, and SP of 93.54, 93.41, and 93.68%, whereas independent datasets achieved ACC, SN, and SP of 88.45, 67.64, and 94.59%, respectively (Arif et al., 2020).

BChemRF-CPPred (beyond chemical rules-based framework for CPP prediction) was an outstanding technique that exploited different sequence- and structure-based descriptors with ANN, SVM, and GPC to differentiate CPPs and non-CPPs from training and independent datasets. The independent datasets achieved a prediction accuracy of 90.66% with SN and SP of 89.30 and 92%, respectively (de Oliveira et al., 2021). A deep-learning interpretable method, ITP-Pred, was developed with feature encodings of AAC and PCP. Convolutional neural networks (CNNs) and recurrent neural networks (RNNs) are types of ANNs, of which long short-term memory (LSTM) is a distinct type of RNN. ITP-Pred utilized the CNN-BiLSTM algorithm, which is a fusion of CNN and LSTM with the feature descriptors and evaluated with a 5-fold strategy. The ACC, SN, and SP of training sets were 89, 86.30, and 93.20%, and the validation sets were 95.10, 92.80, and 97.80%, respectively (Cai et al., 2021).

The prediction and uptake efficiency strength were simultaneously performed with an updated version of MLCPP, a stacking 2-layer approach tool called MLCPP 2.0. The best model was selected from 199 baseline models developed using 7 different ML classifiers (SVM, RF, LGBM, gradient boosting, ADA boosting, XGB, and ERT) and 17 feature encoding algorithms. The thrived ML classifiers were analyzed using a ten-fold assessment method that outperformed other methods, showing ACC, SN, and SP of 91.30, 88.50, and 94.10% on training datasets and 93.40, 84.70, and 94% on independent datasets. To estimate the significance of the top 20 features, an ablation study was performed. A user-friendly web server was implemented for the convenience of researchers (Manavalan and Patra, 2022). DeepCPPred is the first deep learning framework with a two-layer approach followed by an elastic net (EN) algorithm to select appropriate features. Out of the four feature descriptors used, PSSM performed better with remarkable accuracy. The CDF algorithm achieved better prediction results with the PSSM feature (HOG-PSSM) using a 5-fold CV on independent datasets. Layer 1 prediction achieved ACC, SN, and SP of 93.04, 99.34, and 86.73%, and layer 2 predictions achieved 95.43, 95.68, and 95.17%, respectively (Arif et al., 2021).

SiameseCPP is the first tool implemented with a contrastive learning approach for developing an automated CPP prediction model. Siamese neural network (SNN) classifiers used different probabilistic features with a gated recurrent unit (GRU) framework on training datasets. This model was superior to the existing baseline models with ACC, SN, and SP of 96.17, 95.92, and 96.47% (Zhang et al., 2023). With the aim of predicting efficient CPPs, AiCPP was established in a sliding window approach. This is a deep-learning framework that exploited the LSTM algorithm, which is a specialized RNN with the 9-mer approach. AiCPP used many training and test datasets. The model demonstrated a better performance with ACC, SN, and SP of 86, 82.70, and 89.30% with the test sets (Park et al., 2023).

PractiCPP is exclusively designed for incredibly imbalanced datasets. Hard negative sampling and feature extraction with the prediction module were the two elements of this method. SF, LSF, and PTF were the three unique features utilized for model prediction in PractiCPP_base_. The imbalanced dataset was kept at a 1:1000 ratio for evaluating the performance. A 10-fold CV performance evaluation was carried out on balanced datasets from CPP924 datasets. PractiCPP_base_ achieved ACC, SN, and SP of 95.65, 94.29, and 97.06%. Precision, recall, F1 score, and FP/C (FP per correct) for imbalanced datasets were 80.56, 60, 68.64, and 24.14%, respectively. An ablation study was performed to analyze the influence of feature embeddings like pre-trained features and Morgan fingerprints (Shi et al., 2024).

LightCPPgen is a recent predictor that utilized the datasets from MLCPP 2.0 (Manavalan and Patra, 2022). Various sequence- and structure-based features were included from the iFeature Omega and RDKit libraries, respectively. Exclusive feature bundling (EFB) and gradient-based one-side sampling (GOSS) were the two novel strategies that comprised LGBM to enhance efficiency. This technique is an integration of ML and GA (genetic algorithms). Global feature and local feature attributes were implicated to give a comprehensive picture of the impact of features in the model’s prediction. Around 375 features were scrutinized after the MDI approach (mean decrease in impurity). A 10-fold CV assessment was performed on independent datasets that achieved ACC, SN, and SP of 96.20, 69, and 98.10%, respectively (Maroni et al., 2024).

## 5 Analysis of the performance of existing tools for CPP prediction

**Figure 2:**
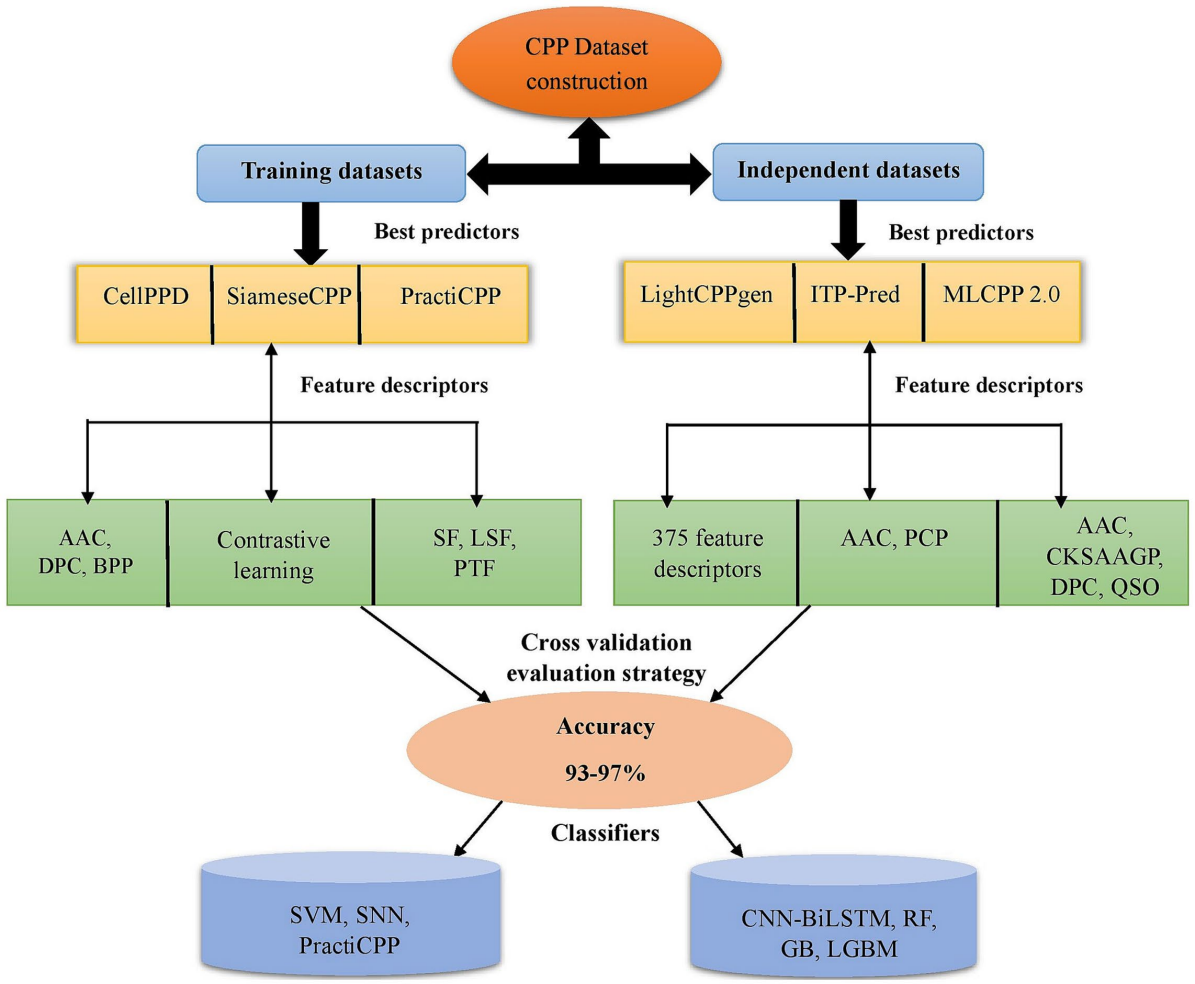
Depiction of existing framework in CPP prediction methods. It represents some of the best predictors on training and independent datasets with feature encodings (AAC, PCP, DPC, CKSAAGP, QSO, etc.) selected for prediction. Different ML classifiers SVM, RF, LGBM, GB, SNN, PractiCPP, and CNN-BiLSTM achieved higher accuracy around 93–97%. The CV technique was utilized for model evaluation. CKSAAGP, composition of k-spaced amino acid group pairs; QSO, quasi sequence order; CV, cross-validation; AAC, amino acid composition; DPC, dipeptide composition; BPP, binary profiles of pattern; PCP, physicochemical properties; SF, sequential features; LSF, local structure features; PTF, pretrained features; GB, gradient boosting; LGBM, light gradient boosting machine; SVM, support vector machine; SNN, siamese neural network; RF, random forest; CNN-BiLSTM, convolutional neural network-bidirectional long short-term memory.

Training datasets serve as the basis for the model for recognizing patterns and correlations within the incoming data. They are used for internal evaluation of the developed model. Independent datasets aid in predicting the robustness of the model. They are crucial for assessing the model’s efficacy and confirming its real-world applicability. Figure 2 depicts the CPP prediction framework via a flowchart of efficient prediction methods in chronological order. MCC and AUC are the assessment metrics crucial for determining the efficiency of prediction, particularly in classification tasks of machine learning. If the MCC and AUC values are closer to 1, the developed ML method achieved robust performance in prediction (Basith et al., 2020).

### 5.1 Comparison of the size of datasets

**Figure 3:**
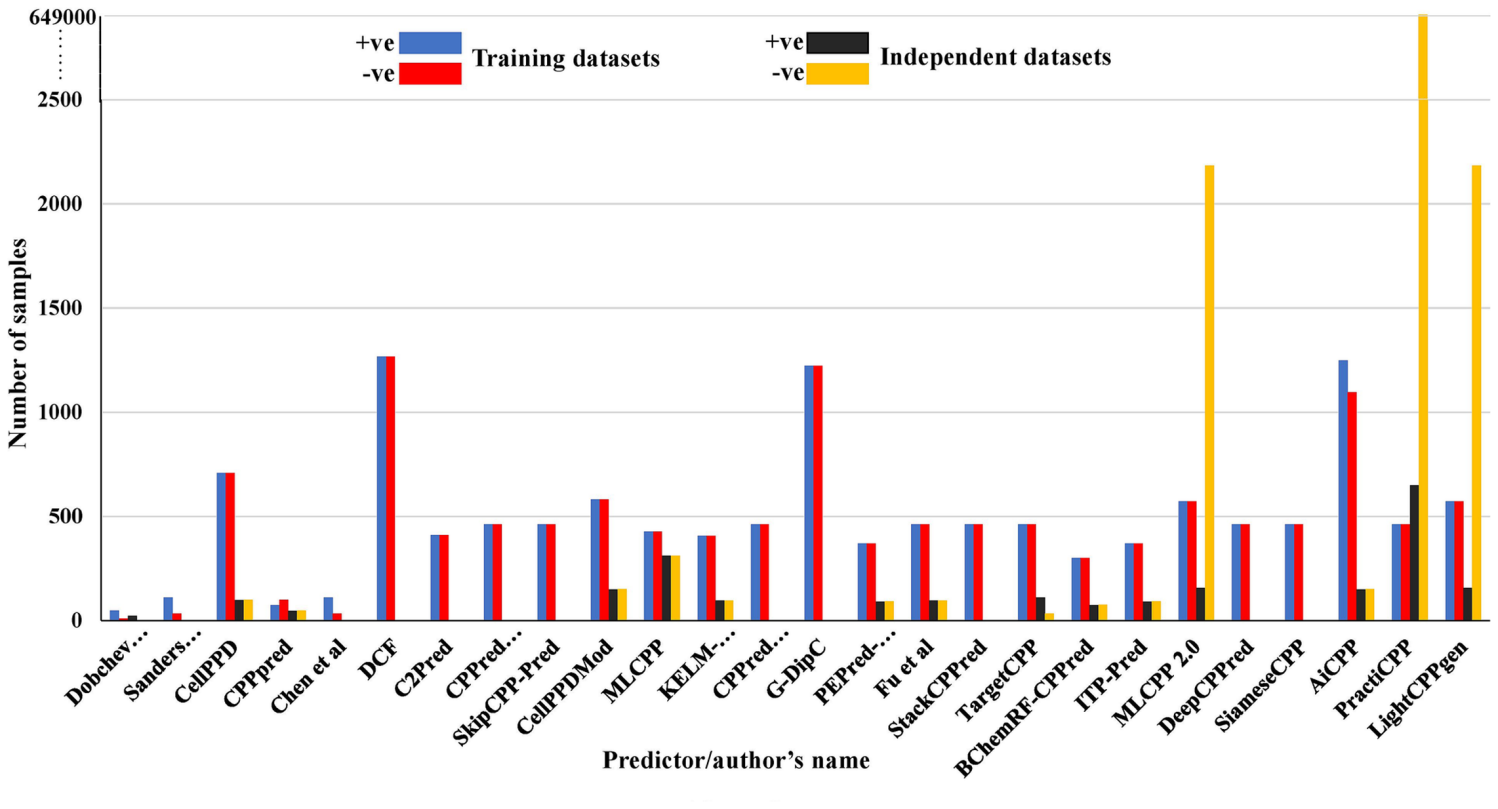
Comparison of size of training and independent datasets used on state-of-the-art methods for CPP prediction.

The size of training and independent datasets for all the prediction methods is represented in Figure 3. CPPs (+ve) and non-CPPs (−ve) were balanced to reduce the overfitting of the model in the majority of the prediction methods. Dobchev utilized the minimum number of training and independent datasets, which contain 59 (49/10) and 25 (23/2) sequences retrieved from available literature, respectively (Dobchev et al., 2010). Sanders utilized 145 (111/34) training datasets, which were unbalanced (Sanders et al., 2011). This dataset was utilized for training the model in Chen et al.’s prediction and testing the prediction in TargetCPP (Chen et al., 2015; Arif et al., 2020). CPPpred used an unbalanced training set (74/100) and a balanced test set (47/47) for model generation (Holton et al., 2013). CellPPD used 198 (99/99) sequences as a validation set (Gautam et al., 2013). MLCPP utilized 854 (427/427) sequences as a training set and 622 (311/311) sequences as a validation set (Manavalan et al., 2018). KELM-CPPpred utilized 816 (408/408) sequences as a training dataset (Pandey et al., 2018). Fu et al.’s predictor and KELM-CPPpred utilized a balanced independent dataset (96/96) for evaluation (Pandey et al., 2018; Fu et al., 2019). PEPred-Suite and ITP-Pred utilized 740 peptides (370/370) for training and 184 peptides (92/92) for validating the prediction (Wei L. et al., 2019; Cai et al., 2021). BChemRF-CPPred used 600 sequences (300/300) for training and 150 sequences (75/75) for testing the prediction (de Oliveira et al., 2021). CPPred-RF (Wei et al., 2017b), SkipCPP-Pred (Wei et al., 2017a), CPPred-FL (Qiang et al., 2020), StackCPPred (Fu et al., 2020), TargetCPP (Arif et al., 2020), MLCPP 2.0 (Manavalan and Patra, 2022), DeepCPPred (Arif et al., 2021), Fu et al.’s predictor (Fu et al., 2019), SiameseCPP (Zhang et al., 2023), and PractiCPP (Shi et al., 2024) are the ten predictors that retrieved the datasets from CPP924 (Wei et al., 2017b). CellPPD (Gautam et al., 2013), CPPred (Holton et al., 2013), CellPPD-Mod (Kumar et al., 2018), MLCPP (Manavalan et al., 2018), KELM-CPPpred (Pandey et al., 2018), PEPred-Suite (Wei L. et al., 2019), Fu et al.’s predictor (Fu et al., 2019), BChemRF-CPPred (de Oliveira et al., 2021), and ITP-Pred (Cai et al., 2021) are the nine predictors where both training and independent datasets are balanced. C2Pred used 822 (411/411) sequences as a training set (Tang et al., 2016). CellPPD-Mod and AiCPP utilized the same number of balanced independent datasets (150/150) (Kumar et al., 2018; Park et al., 2023). DCF (Diener et al., 2016) used 2,534 (1,265 CPPs and 1,265 non-CPPs), the maximum number of training datasets, followed by G-DipC (Wang et al., 2019), AiCPP (Park et al., 2023), CellPPD (Gautam et al., 2013), and CellPPD-Mod (Kumar et al., 2018) with training datasets of 2,446 (1,223/1223), 2,346 (249/1097), 1,416 (708/708), and 1,164 (582/582). AiCPP utilized a comparatively larger, unbalanced training dataset among all 26 predictors (Park et al., 2023). MLCPP 2.0 used 1,146 (573/573) and 2,341 (157/2184) balanced training and unbalanced independent datasets for model prediction, respectively (Manavalan and Patra, 2022). LightCPPgen retrieved the datasets from MLCPP 2.0 (layer-1) for prediction and validation (Maroni et al., 2024). PractiCPP was exclusively developed for imbalanced datasets. This method involved the balanced datasets from CPP924 (462/462) for reasonable comparison with existing predictors. A 1:1000 ratio of the imbalanced dataset (649/649000) was included for prediction, which performed well with good precision and specificity. This is considered the largest among all the datasets (Shi et al., 2024). Sanders et al.’s predictor (Sanders et al., 2011), Chen et al.’s predictor (Chen et al., 2015), DCF (Diener et al., 2016), C2Pred (Tang et al., 2016), SkipCPP-Pred (Wei et al., 2017a), CPPred-RF (Wei et al., 2017b), CPPred-FL (Qiang et al., 2020), G-DipC (Wang et al., 2019), StackCPPred (Fu et al., 2020), DeepCPPpred (Arif et al., 2021), and SiameseCPP (Zhang et al., 2023) are the eleven predictors that did not include an independent dataset for external validation. Five predictors followed a 2-layer prediction framework in which CPPred-RF (Wei et al., 2017b), MLCPP (Manavalan et al., 2018), StackCPPred (Fu et al., 2020), and DeepCPPred (Arif et al., 2021) utilized a balanced dataset (187/187) for the estimation of uptake efficiency retrieved from CPPsite3 (Wei et al., 2017b). MLCPP 2.0 (Manavalan and Patra, 2022) utilized 46 high-uptake and 16 low-uptake CPPs from the MLCPP dataset and CPPsite 2.0 (Manavalan et al., 2018). CPPsite 2.0 is a database with around 1855 experimentally validated peptide entries from which CPPs can be retrieved and utilized for research (Agrawal et al., 2016).

### 5.2 Evaluation of the performance of training datasets

The experimental and statistical results of existing prediction methods on training datasets are presented in Table 2. Fifteen predictors achieved an accuracy (ACC) range of 90 to 97%. Nine predictors achieved an ACC range of 77 to 89%. DCF, Dochev’s predictor, and CPPred reported only ACC, while other assessment metrics SN, SP, MCC, and AUC with training datasets were not indicated properly (Diener et al., 2016; Dobchev et al., 2010; Holton et al., 2013). The statistical results of the training set were not reported in AiCPP and LightCPPgen (Park et al., 2023; Maroni et al., 2024). The MCC value of four predictors, CellPPD (Gautam et al., 2013), SiameseCPP (Zhang et al., 2023), PractiCPP (Shi et al., 2024), and CellPPD-Mod (Kumar et al., 2018), is above 0.9, indicating perfect prediction. Nine predictors achieved an MCC range between 0.81 and 0.89, indicating better prediction. Four predictors achieved an average MCC range between 0.71 and 0.78, indicating moderate prediction. Chen et al.’s predictor obtained an MCC of 0.486, which achieved poor prediction performance (Chen et al., 2015). C2Pred and Sanders et al.’s predictor estimated only three statistical metrics: ACC, SN, and SP (Tang et al., 2016; Sanders et al., 2011). Eleven predictors estimated the AUC range around 0.92 to 0.99, indicating a perfect classifier in prediction. The AUC is one of the important evaluation metrics that was not reported in thirteen predictors, namely, SiameseCPP (Zhang et al., 2023), PractiCPP (Shi et al., 2024), StackCPPred (Fu et al., 2020), TargetCPP (Arif et al., 2020), Sanders et al.’s predictor (Sanders et al., 2011), SkipCPP-Pred (Wei et al., 2017a), DCF (Diener et al., 2016), BChemRF-CPPred (de Oliveira et al., 2021), G-DipC (Wang et al., 2019), C2Pred (Tang et al., 2016), Chen et al.’s predictor (Chen et al., 2015), Dobchev et al.’s predictor (Dobchev et al., 2010), and CPPpred (Holton et al., 2013). CellPPD (Gautam et al., 2013), CellPPD-Mod (Kumar et al., 2018), and DeepCPPred (Arif et al., 2021) achieved the maximum AUC of 0.99 from the ROC curve, indicating remarkable predictivity. Fourteen predictors reported a higher precision (SN) range between 90 and 98%. Five predictors estimated a moderate SN range between 81 and 89%. G-DipC reported the lowest SN of 65.28%, which is the least preferred method for prediction (Wang et al., 2019). Thirteen predictors recorded higher recall (SP) ranges between 91 and 97%. Four predictors estimated the average SP range between 84 and 90%. G-DipC estimated moderate SP of 70.67% (Wang et al., 2019). Chen et al.’s predictor estimated a poor SP of 44.10%, whereas the SN is higher at 95.50% (Chen et al., 2015). Sensitivity and specificity were not reported in DCF (Diener et al., 2016), BChemRF-CPPred (de Oliveira et al., 2021), Dobchev et al.’s predictor (Dobchev et al., 2010), and CPPpred (Holton et al., 2013). Hence, random accuracy was obtained, and effectiveness was not defined in these predictors.

**Table 2:** Comparison of available prediction methods on training datasets evaluated with the cross-validation technique.

S No	Predictors	ACC (%)	SN (%)	SP (%)	MCC	AUC	References
1	CellPPD	97.40	98.15	96.58	0.950	0.990	Gautam et al. (2013)
2	SiameseCPP	96.17	95.92	96.47	0.923	NR	Zhang et al. (2023)
3	PractiCPP	95.65	94.29	97.06	0.913	NR	Shi et al. (2024)
4	CellPPD-Mod	95.10	95.19	95.02	0.900	0.990	Kumar et al. (2018)
5	StackCPPred	94.50	94.20	94.80	0.890	NR	Fu et al. (2020)
6	TargetCPP	93.54	93.41	93.68	0.871	NR	Arif et al. (2020)
7	DeepCPPred	93.04	99.34	86.73	0.878	0.993	Arif et al. (2021)
8	Fu et al.	92.30	91.80	92.90	0.846	0.957	Fu et al. (2019)
9	CPPred-FL	92.10	92.40	91.80	0.842	0.976	Qiang et al. (2020)
10	Sanders et al.	91.72	91.70	12.70	NR	NR	Sanders et al. (2011)
11	CPPred-RF	91.60	90.50	92.60	0.831	0.972	Wei et al. (2017b)
12	MLCPP 2.0	91.30	88.50	94.10	0.827	0.949	Manavalan and Patra (2022)
13	PEPred-Suite	91.20	90.30	92.20	0.824	0.972	Wei L. et al. (2019)
14	SkipCPP-Pred	90.60	88.50	92.60	0.812	NR	Wei et al. (2017a)
15	DCF	90.00	NR	NR	NR	NR	Diener et al. (2016)
16	ITP-Pred	89.00	86.30	93.20	0.787	0.962	Cai et al. (2021)
17	MLCPP	88.30	91.90	84.50	0.768	0.938	Manavalan et al. (2018)
18	BChemRF-CPPred	87.60	NR	NR	NR	NR	de Oliveira et al. (2021)
19	KELM-CPPpred	86.21	82.61	89.56	0.730	0.920	Pandey et al. (2018)
20	G-DipC	83.98	65.28	70.67	0.712	NR	Wang et al. (2019)
21	C2Pred	83.60	81.50	85.60	NR	NR	Tang et al. (2016)
22	Chen et al.	83.40	95.50	44.10	0.486	NR	Chen et al. (2015)
23	Dobchev et al.	83.10	NR	NR	NR	NR	Dobchev et al. (2010)
24	CPPpred	77.60	NR	NR	NR	NR	Holton et al. (2013)

NR, not reported; ACC, accuracy; SN, sensitivity; SP, specificity; MCC, Matthews correlation coefficient; AUC, area under the curve.

**Figure 4:**
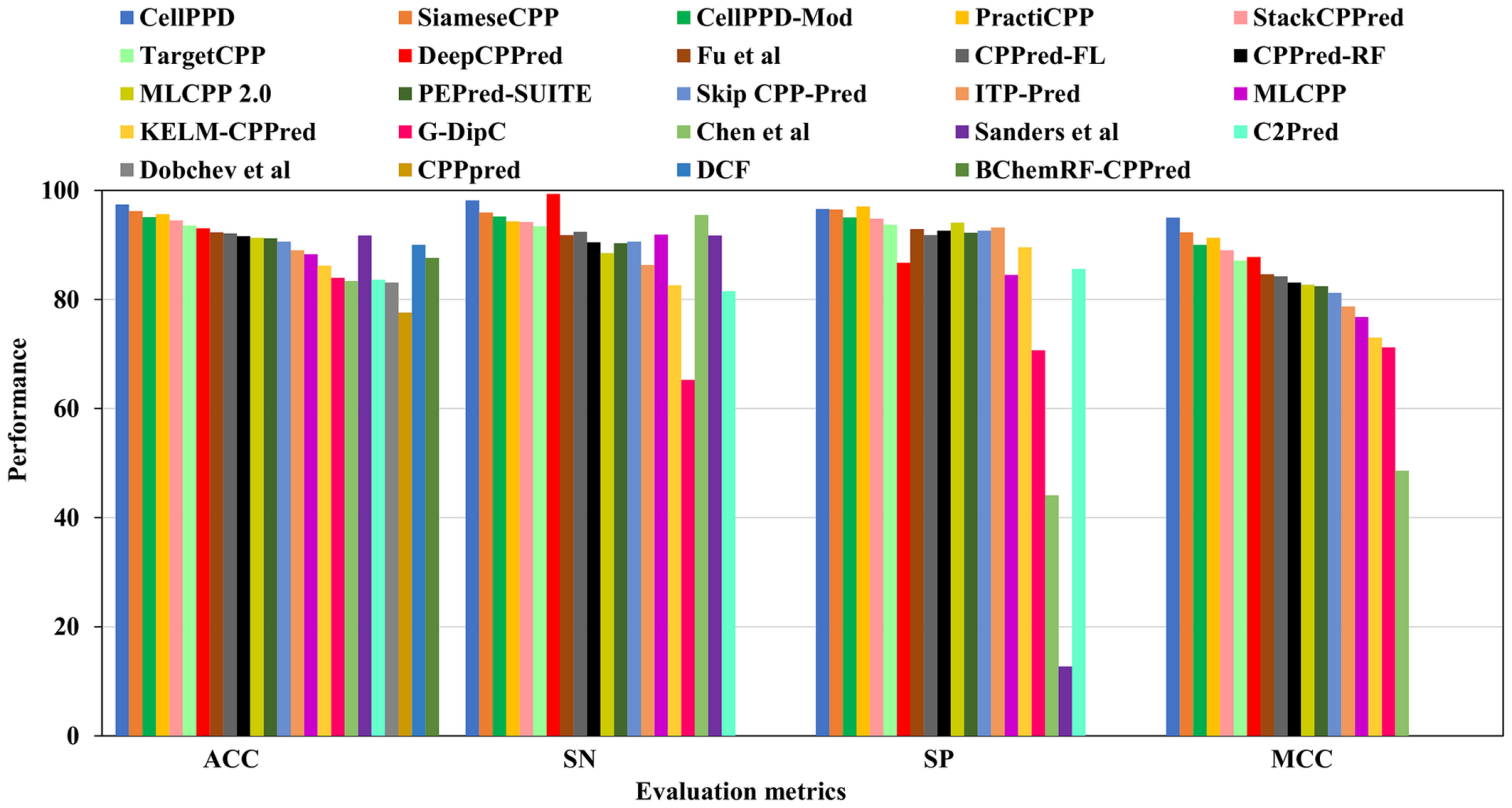
Comparison results of existing CPP prediction methods on training datasets. Accuracy (ACC), sensitivity (SN), specificity (SP), and Matthews correlation coefficient (MCC).

CellPPD achieved the best results in prediction with 97.40% ACC and 0.950 MCC (Gautam et al., 2013). The sensitivity (SN) is 1.19% lower than DeepCPPred, which achieved the best precision of 99.34% (Arif et al., 2021). However, the SN is 2.23, 2.65, and 2.96% higher than SiameseCPP (Zhang et al., 2023), Chen et al.’s predictor (Chen et al., 2015), and CellPPD-Mod (Kumar et al., 2018). The specificity (SP) is 0.48% lower than PractiCPP, which achieved the best recall score of 97.06% among all the predictors (Shi et al., 2024). However, the SP is 0.11, 1.56, and 1.78% higher than SiameseCPP (Zhang et al., 2023), CellPPD-Mod (Kumar et al., 2018), and StackCPPred (Fu et al., 2020). SiameseCPP is the second-best predictor, with 96.17% ACC and 0.923 MCC (Zhang et al., 2023). PractiCPP is the third-best predictor with 95.65% ACC and 0.913 MCC (Shi et al., 2024). CellPPD-Mod follows PractiCPP with 95.10% ACC and 0.90 MCC (Kumar et al., 2018). StackCPPred achieved better prediction, the 5th best, with ACC and MCC of 94.50% and 0.890, respectively (Fu et al., 2020). TargetCPP achieved 93.54% ACC and 0.871 MCC, the 6th best in predicting efficient CPPs (Arif et al., 2020). MCC values of DeepCPPred (Arif et al., 2021), Fu et al.’s predictor (Fu et al., 2019), CPPred-FL (Qiang et al., 2020), CPPred-RF (Wei et al., 2017b), MLCPP 2.0 (Manavalan and Patra, 2022), PEPred-Suite (Wei L. et al., 2019), and SkipCPP-Pred (Wei et al., 2017a) were 0.878, 0.846, 0.842, 0.831, 0.827, 0.824, and 0.812. These predictors achieved better efficiency in predicting CPPs. ITP-Pred (Cai et al., 2021), MLCPP (Manavalan et al., 2018), KELM-CPPpred (Pandey et al., 2018), AiCPP (Park et al., 2023), and G-DipC (Wang et al., 2019) achieved moderate performance. A pictorial representation of different evaluation metrics for the available CPP prediction methods CellPPD (Gautam et al., 2013), SiameseCPP (Zhang et al., 2023), PractiCPP (Shi et al., 2024), CellPPD-Mod (Kumar et al., 2018), StackCPPred (Fu et al., 2020), TargetCPP (Arif et al., 2020), DeepCPPred (Arif et al., 2021), Fu et al.’s predictor (Fu et al., 2019), CPPred-FL (Qiang et al., 2020), Sanders et al.’s predictor (Sanders et al., 2011), CPPred-RF (Wei et al., 2017b), MLCPP 2.0 (Manavalan and Patra, 2022), PEPred-Suite (Wei L. et al., 2019), and SkipCPP-Pred (Wei et al., 2017a), DCF (Diener et al., 2016), ITP-Pred (Cai et al., 2021), MLCPP (Manavalan et al., 2018), BChemRF-CPPred (de Oliveira et al., 2021), KELM-CPPpred (Pandey et al., 2018), G-DipC (Wang et al., 2019), C2Pred (Tang et al., 2016), Chen et al.’s predictor (Chen et al., 2015), Dobchev et al.’s predictor (Dobchev et al., 2010), and CPPpred (Holton et al., 2013) on training datasets are depicted in Figure 4. AUC values of CPPred-RF (Qiang et al., 2020), MLCPP (Manavalan et al., 2018), KELM-CPPpred (Pandey et al., 2018), CPPred-FL (Qiang et al., 2020), PEPred-Suite (Wei L. et al., 2019), ITP-Pred (Cai et al., 2021), MLCPP 2.0 (Manavalan and Patra, 2022), DeepCPPred (Arif et al., 2021), Fu et al.’s predictor (Fu et al., 2019), and AiCPP (Park et al., 2023) were 0.972, 0.938, 0.920, 0.976, 0.972, 0.962, 0.949, 0.993, 0.957, and 0.927, indicating robustness of the prediction. SVM (Kumar et al., 2018), SNN (Zhang et al., 2023), and RF (Kumar et al., 2018) are some ML algorithms implemented by the predictors that accomplished the best performance on training datasets in determining efficient CPPs. In PractiCPP, the hard negative sampling contributed to the better classification of CPP in the imbalanced dataset, which performed greater than the variant PractiCPP_base_ (Shi et al., 2024).

### 5.3 Evaluation of the performance of independent datasets

Overfitting of the predictive model may occur while striving to achieve the maximum MCC or accuracy during training. An independent review of the established model is a prerequisite for mitigating such presumptions. Table 3 elucidated the comparison of existing prediction methods on independent datasets. 15 out of 26 predictors calculated the effectiveness of predictions using test datasets. Six predictors, LightCPPgen (Maroni et al., 2024), ITP-Pred (Cai et al., 2021), MLCPP 2.0 (Manavalan and Patra, 2022), CellPPD-Mod (Kumar et al., 2018), Dobchev et al.’s predictor (Dobchev et al., 2010), and BChemRF-CPPred (de Oliveira et al., 2021), accomplished a higher ACC range between 90 and 96%. Seven predictors, MLCPP (Manavalan et al., 2018), TargetCPP (Arif et al., 2020), AiCPP (Park et al., 2023), Fu et al.’s predictor (Fu et al., 2019), KELM-CPPpred (Pandey et al., 2018), CPPred (Holton et al., 2013), and CellPPD (Gautam et al., 2013), indicated an average ACC range between 81 and 89%. ITP-Pred is the only predictor with a remarkable MCC value, indicating perfect prediction suitable for real-world application (Cai et al., 2021). CellPPD-Mod (Kumar et al., 2018) and BChemRF-CPPred (de Oliveira et al., 2021) achieved an average MCC value above 0.80. MLCPP and AiCPP indicated a moderate MCC value above 0.70 (Manavalan et al., 2018; Park et al., 2023). Six predictors, LightCPPgen (Maroni et al., 2024), MLCPP 2.0 (Manavalan and Patra, 2022), TargetCPP (Arif et al., 2020), Fu et al.’s predictor (Fu et al., 2019), KELM-CPPpred (Pandey et al., 2018), and CellPPD (Gautam et al., 2013), scored a moderate MCC value between 0.62 and 0.69. The MCC value was not reported in four predictors: Dobchev et al.’s predictor (Dobchev et al., 2010), CPPred (Holton et al., 2013), PEPred-Suite (Wei L. et al., 2019), and PractiCPP (Shi et al., 2024). Nine predictors estimated the AUC range around 0.91 to 0.99, indicating a perfect classifier in the validation of test datasets. F1 score was one of the evaluation metrics reported in PractiCPP with 0.686 indicating an average efficiency in CPP determination. However, PractiCPP reported a moderate AUC of 0.64, indicating mediocre discrimination ability (Shi et al., 2024). Eleven predictors reported SN and SP in external validation. Three predictors, MLCPP (Manavalan et al., 2018), ITP-Pred (Cai et al., 2021), and CellPPD-Mod (Kumar et al., 2018), estimated higher SN of above 90%. Five predictors, MLCPP 2.0 (Manavalan and Patra, 2022), BChemRF-CPPred (de Oliveira et al., 2021), AiCPP (Park et al., 2023), Fu et al.’s predictor (Fu et al., 2019), and PractiCPP (Shi et al., 2024), recorded an average SN range between 80 and 89%. Three predictors, LightCPPgen (Maroni et al., 2024), TargetCPP (Arif et al., 2020), and KELM-CPPpred (Pandey et al., 2018), reported a moderate SN of below 80%. Six predictors, LightCPPgen (Maroni et al., 2024), ITP-Pred (Cai et al., 2021), MLCPP 2.0 (Manavalan and Patra, 2022), CellPPD-Mod (Kumar et al., 2018), BChemRF-CPPred (de Oliveira et al., 2021), and TargetCPP (Arif et al., 2020), attained an outstanding SP range between 92 and 98%. Four predictors, MLCPP (Manavalan et al., 2018), AiCPP (Park et al., 2023), Fu et al.’s predictor (Fu et al., 2019), and KELM-CPPpred (Pandey et al., 2018), recorded an average SP range of above 80%. PractiCPP achieved a lower SP of 60% in the estimation of effective CPPs (Shi et al., 2024). Four predictors—Dobchev et al.’s predictor (Dobchev et al., 2010), CPPred (Holton et al., 2013), CellPPD (Gautam et al., 2013), and PEPred-Suite (Wei L. et al., 2019)—have not reported precision (SN) and recall (SP), which are the important statistical metrics for CPP discrimination.

**Table 3:** Comparison of available prediction methods on independent datasets evaluated with the CV strategy.

S no	Predictors	ACC (%)	SN (%)	SP (%)	MCC	AUC	References
1	LightCPPgen	96.20	69.00	98.10	0.687	0.930	Maroni et al. (2024)
2	ITP-Pred	95.10	92.80	97.80	0.904	0.989	Cai et al. (2021)
3	MLCPP 2.0	93.40	84.70	94.00	0.624	0.928	Manavalan and Patra (2022)
4	CellPPD-Mod	92.33	91.33	93.33	0.850	0.980	Kumar et al. (2018)
5	Dobchev et al.	92.00	NR	NR	NR	NR	Dobchev et al. (2010)
6	BChemRF-CPPred	90.66	89.30	92.00	0.813	0.953	de Oliveira et al. (2021)
7	MLCPP	89.60	93.30	85.80	0.793	0.959	Manavalan et al. (2018)
8	TargetCPP	88.45	67.64	94.59	0.675	NR	Arif et al. (2020)
9	AiCPP	86.00	82.70	89.30	0.722	0.927	Park et al. (2023)
10	Fu et al.	84.38	82.29	86.46	0.688	NR	Fu et al. (2019)
11	KELM-CPPpred	83.10	78.72	88.05	0.670	0.910	Pandey et al. (2018)
12	CPPred	82.98	NR	NR	NR	NR	Holton et al. (2013)
13	CellPPD	81.30	NR	NR	0.630	NR	Gautam et al. (2013)
14	PEPred-Suite	NR	NR	NR	NR	0.952	Wei L. et al. (2019)
15	PractiCPP	NR	80.56	60.00	NR	0.640	Shi et al. (2024)

NR, not reported; ACC, accuracy; SN, sensitivity; SP, specificity; MCC, Matthews correlation coefficient; AUC, area under the curve.

**Figure 5:**
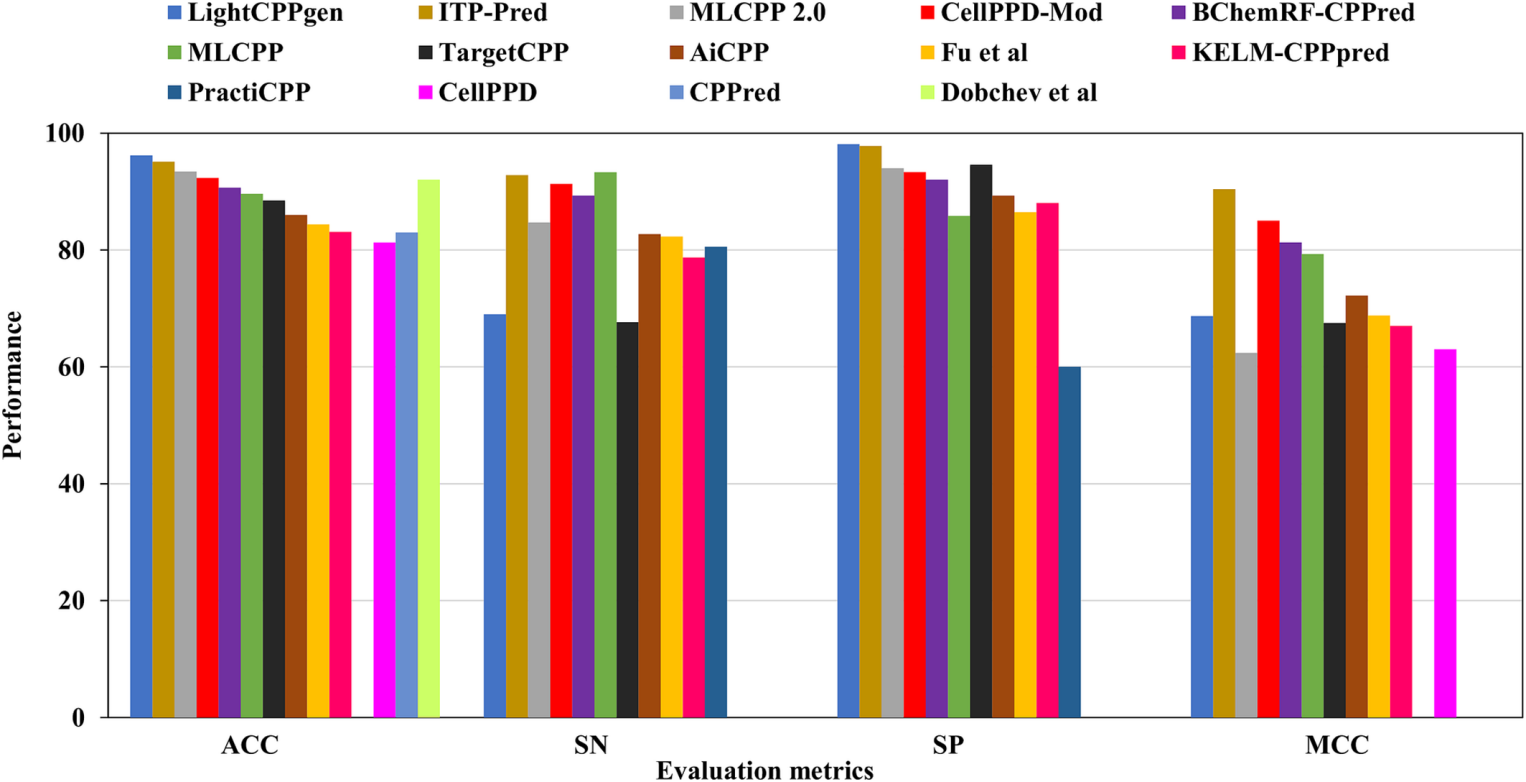
Comparison results of existing CPP prediction methods on independent datasets. Accuracy (ACC), sensitivity (SN), specificity (SP), and Matthews correlation coefficient (MCC).

LightCPPgen accomplished a remarkable ACC of 96.20% and an AUC of 0.93. MCC of 0.687 indicated moderate predictive ability (Maroni et al., 2024). The SN is only 69%, which is 24.3, 23.8, 22.3, 20.3, and 15.7% lower than MLCPP (Manavalan et al., 2018), ITP-Pred (Cai et al., 2021), CellPPD-Mod (Kumar et al., 2018), BChemRF-CPPred (de Oliveira et al., 2021), and MLCPP 2.0 (Manavalan and Patra, 2022). The SP is 98.10%, which is 0.30, 3.51, 4.10, and 4.77% higher than ITP-Pred (Cai et al., 2021), TargetCPP (Arif et al., 2020), MLCPP 2.0 (Manavalan and Patra, 2022), and CellPPD-Mod (Kumar et al., 2018), indicating its precise prediction of negative cases. ITP-Pred achieved outstanding predictive ability with ACC, MCC, and AUC of 95.10%, 0.904, and 0.989 (Cai et al., 2021). MLCPP 2.0 achieved better predictive ability with ACC, MCC, and AUC of 93.40%, 0.624, and 0.928 (Manavalan and Patra, 2022). CellPPD-Mod is the fourth among the top predictors that achieved a good performance with ACC, MCC, and AUC of 92.33%, 0.850, and 0.98 (Kumar et al., 2018). BChemRF-CPPred achieved ACC, MCC, and AUC of 90.66%, 0.813, and 0.953 (de Oliveira et al., 2021). MCC values of MLCPP (Manavalan et al., 2018), TargetCPP (Arif et al., 2020), AiCPP (Park et al., 2023), Fu et al.’s predictor (Fu et al., 2019), KELM-CPPpred (Pandey et al., 2018), and CellPPD (Gautam et al., 2013) were 0.793, 0.675, 0.722, 0.688, 0.670, and 0.630. AUC values of MLCPP (Manavalan et al., 2018), KELM-CPPpred (Pandey et al., 2018), PEPred-Suite (Wei L. et al., 2019), and AiCPP (Park et al., 2023) were 0.959, 0.910, 0.952, and 0.927, indicating the robustness of the predicted model. Predictors with MCC < 0.70 can be improved by varying the hyperparameters to achieve better predictive ability. The top predictors utilized ML algorithms like LGBM (Maroni et al., 2024), RF (Kumar et al., 2018), GB (Manavalan and Patra, 2022), and CNN-BiLSTM (Cai et al., 2021) that achieved remarkable predictions with independent datasets. A pictorial representation of different evaluation metrics for the available CPP prediction methods—LightCPPgen (Maroni et al., 2024), ITP-Pred (Cai et al., 2021), MLCPP 2.0 (Manavalan and Patra, 2022), CellPPD-Mod (Kumar et al., 2018), Dobchev et al.’s predictor (Dobchev et al., 2010), BChemRF-CPPred (de Oliveira et al., 2021), MLCPP (Manavalan et al., 2018), TargetCPP (Arif et al., 2020), AiCPP (Park et al., 2023), Fu et al.’s predictor (Fu et al., 2019), KELM-CPPpred (Pandey et al., 2018), CPPpred (Holton et al., 2013), CellPPD (Gautam et al., 2013), and PractiCPP (Shi et al., 2024) on independent datasets are depicted in Figure 5. The top three prediction methods on training and independent datasets for effective CPP prediction are elucidated in Table 4 to give a deeper understanding of suitable predictors.

**Table 4:** Top 3 CPP prediction methods on training and independent datasets.

S No	Predictors (training datasets)	ACC (%)	SN (%)	SP (%)	MCC	AUC	References
1	CellPPD	97.40	98.15	96.58	0.950	0.990	Gautam et al. (2013)
2	SiameseCPP	96.17	95.92	96.47	0.923	NR	Zhang et al. (2023)
3	PractiCPP	95.65	94.29	97.06	0.913	NR	Shi et al. (2024)

S No	Predictors (independent datasets)	ACC (%)	SN (%)	SP (%)	MCC	AUC	References
1	LightCPPgen	96.20	69.00	98.10	0.687	0.930	Maroni et al. (2024)
2	ITP-Pred	95.10	92.80	97.80	0.904	0.989	Cai et al. (2021)
3	MLCPP 2.0	93.40	84.70	64.00	0.624	0.928	Manavalan and Patra (2022)

NR, not reported; ACC, accuracy; SN, sensitivity; SP, specificity; MCC, Matthews correlation coefficient; AUC, area under the curve.

### 5.4 Comparison of 2-layer framework prediction methods

A two-layer prediction framework was first implemented in CPPred-RF to determine CPPs and their uptake efficiency (Wei et al., 2017b). Table 5 highlights the comparison of statistical results of five different predictors that followed a two-layer approach. CPPred-RF (Wei et al., 2017b), MLCPP (Manavalan et al., 2018), StackCPPred (Fu et al., 2020), MLCPP 2.0 (Manavalan and Patra, 2022), and DeepCPPred (Arif et al., 2021) followed this strategy. In Layer 1 prediction, StackCPPred achieved outstanding performance in determining CPPs with ACC and MCC of 94.50% and 0.890, respectively (Fu et al., 2020). The SN of StackCPPred is 94.20%, which is 2.30, 3.70, and 5.70% higher than MLCPP (Manavalan et al., 2018), CPPred-RF (Wei et al., 2017b), and MLCPP 2.0 (Manavalan and Patra, 2022), but 5.14% lower than DeepCPPred, which achieved a higher sensitivity of 99.34% (Arif et al., 2021). The SP of StackCPPred is 94.80%, which is 0.70, 2.2, 8.07, and 10.30% higher than MLCPP 2.0 (Manavalan and Patra, 2022), CPPred-RF (Wei et al., 2017b), DeepCPPred (Arif et al., 2021), and MLCPP (Manavalan et al., 2018). The AUC was not reported in StackCPPred. DeepCPPred achieved good results with ACC, MCC, and AUC of 93.04%, 0.878, and 0.993 (Arif et al., 2021). CPPred-RF obtained better prediction in finding CPPs with ACC and MCC of 91.60% and 0.831, respectively (Wei et al., 2017b). MLCPP 2.0 obtained MCC and AUC of 0.827 and 0.949, respectively (Manavalan and Patra, 2022). MLCPP obtained MCC and AUC of 0.768 and 0.938, respectively, indicating the random performance of the model (Manavalan et al., 2018).

**Table 5:** Comparison of two-layer prediction methods for cell-penetrating peptides.

S No	Predictors (layer 1)	ACC (%)	SN (%)	SP (%)	MCC	AUC	References
1	StackCPPred	94.50	94.20	94.80	0.890	NR	Fu et al. (2020)
2	DeepCPPred	93.04	99.34	86.73	0.878	0.993	Arif et al. (2021)
3	CPPred-RF	91.60	90.50	92.60	0.831	0.972	Wei et al. (2017b)
4	MLCPP 2.0	91.30	88.50	94.10	0.827	0.949	Manavalan and Patra (2022)
5	MLCPP	88.30	91.90	84.50	0.768	0.938	Manavalan et al. (2018)

S No	Predictors (layer 2)	ACC (%)	SN (%)	SP (%)	MCC	AUC	References
1	DeepCPPred	95.43	95.68	95.17	0.910	0.984	Arif et al. (2021)
2	StackCPPred	78.30	79.10	77.50	0.567	0.802	Fu et al. (2020)
3	MLCPP 2.0	76.80	76.40	77.10	0.536	0.824	Manavalan and Patra (2022)
4	MLCPP	72.50	71.70	73.30	0.445	0.764	Manavalan et al. (2018)
5	CPPred-RF	71.10	72.20	70.10	0.423	NR	Wei et al. (2017b)

NR, not reported; ACC, accuracy; SN, sensitivity; SP, specificity; MCC, Matthews correlation coefficient; AUC, area under the curve.

In layer 2 prediction, DeepCPPred achieved the highest accuracy in evaluating the uptake efficiency of CPPs. It achieved ACC, MCC, and AUC of 95.43%, 0.910, and 0.984, indicating outstanding performance (Arif et al., 2021). It accomplished a remarkable SN of 95.68%, which is 16.58, 19.28, 23.48, and 23.98% higher than StackCPPred (Fu et al., 2020), MLCPP 2.0 (Manavalan and Patra, 2022), CPPred-RF (Wei et al., 2017b), and MLCPP (Manavalan et al., 2018). The SP of DeepCPPred is 95.17%, which is 17.67, 18.07, 21.87, and 25.07% higher than StackCPPred (Fu et al., 2020), MLCPP 2.0 (Manavalan and Patra, 2022), MLCPP (Manavalan et al., 2018), and CPPred-RF (Wei et al., 2017b). StackCPPred (Fu et al., 2020) and MLCPP 2.0 (Manavalan and Patra, 2022) achieved moderate performance with ACC of 78.30% and 76.80%, respectively. The MCC value is around 0.5, which indicates random performance of the model. MLCPP (Manavalan et al., 2018) and CPPred-RF (Wei et al., 2017b) achieved average performance with ACC, SN, and SP compared to other predictors in determining the uptake efficiency. The AUC was not reported in CPPred-RF. The MCC value is comparatively lower for MLCPP (0.445) and CPPred-RF (0.423), indicating the least preference of the predictors for finding the uptake efficiency (Manavalan et al., 2018; Wei et al., 2017b). Hyperparameter optimization should be carried out to improve their effectiveness in prediction. Overall, DeepCPPred is appropriate for a 2-layer prediction framework since it achieved exceptional results in all evaluation metrics.

## 6 Common limitations of the CPP prediction methods

Despite significant advancements in forecasting CPPs, various fields remain to be explored. The limited length of CPPs hinders the extraction of contextually disguised information that elucidates their intrinsic properties. The exploration of limited feature representation is a vital factor, and integration of many variables from different domains was conducted to incorporate essential probabilistic information for prediction. This may result in several issues, including the time required to create predictive models and the curse of dimensionality in predictions (Fu et al., 2020). CPPred-FL processed this by employing mRMR to reduce the dimensionality of the feature space (Wei et al., 2017b). Of the 26 predictors examined in this study, 21 can distinguish actual CPPs from non-CPPs. The prospective therapeutic use of CPPs is intricately linked to their absorption efficiency. Prediction of the uptake efficiency involves only 5 predictors. It is essential to anticipate more prediction methods for determining absorption efficiency to uncover the significant potential of CPPs in future therapeutic applications. Three predictors, namely, DeepCPPred, AiCPP, and PractiCPP, utilized the deep-learning technique to make predictions (Arif et al., 2021; Park et al., 2023; Shi et al., 2024). Therefore, the efficacy of CPP utilizing deep-learning methodologies needs more examination. A prevalent drawback is the overfitting of training data, resulting in bias during model building, which poses a significant problem in the absence of a test dataset for external evaluation. Concerning the reliability of machine learning findings, reiterating the evaluation metrics is challenging unless the machine learning conditions and appropriate source codes for feature encodings are well characterized. Therefore, it is essential to supply source codes and data sets for the built methods, facilitating the advancement of next-generation tools (Basith et al., 2020).

## 7 Discussion

From the comparative analysis, CellPPD achieved exceptional performance with good precision on training datasets, while CPPred achieved a moderate accuracy in prediction. In CellPPD, the binary pattern profile feature played a significant role in the excellent predictive ability with the SVM algorithm (Gautam et al., 2013). A crystal-clear web tool was created for biological aspirants to carry out research in designing effective CPPs. LightCPPgen was able to achieve the highest performance on independent datasets with remarkable accuracy and sensitivity (Maroni et al., 2024). However, MLCPP performed better with the highest precision (Manavalan et al., 2018). In ITP-Pred, AAC and PCP were the feature descriptors that contributed to the robustness of the model generated with the CNN-BiLSTM algorithm that achieved the highest MCC and AUC (Cai et al., 2021). StackCPPred and DeepCPPred demonstrated high precision in Layer 2 prediction (Fu et al., 2020; Arif et al., 2021). This helped in determining effective CPPs with better uptake efficiency from large-scale datasets. The HOG-PSSM feature contributed to the robust performance of DeepCPPred (Arif et al., 2021). The development of more refined target-specific drug delivery systems employing CPPs with fewer adverse effects must be investigated to address issues that prevent their practical utility. PractiCPP is one of the several deep learning frameworks that offered a promising solution to address the challenges posed by imbalanced binary classification in CPP prediction. The model’s propensity to handle imbalanced data and its state-of-the-art performance on balanced and imbalanced datasets denote its capacity for practical deployment in drug delivery research and development (Shi et al., 2024). In addition, it compels the model to focus on challenging negative samples, refining its decision boundaries, and augmenting its overall performance. By adapting the framework of PractiCPP, we may be able to evolve novel strategies to overcome challenges in various prediction methods in computational biology. In conclusion, we anticipate that no single model consistently surpasses all others in every assessment parameter.

## 8 Conclusion and future prospects

We have reviewed 26 prediction methods using ML algorithms and deep-learning techniques regarding statistical metrics, feature encodings, and dataset size. Furthermore, we have discussed their importance in therapeutics and their limitations. ML predictions must be validated experimentally. A thorough understanding of the biological processes behind CPPs helps the researchers find out specific assays for targeting specific cell types for delivering cargo molecules. Mechanistic understanding guarantees that predictions are physiologically relevant, interpretable, and actionable. This facilitates the effective identification and creation of new CPPs with therapeutic potential. Although excellent results have been obtained from these ML-oriented in silico tools, additional studies are essential for a thorough understanding. Only a few ML methods have been evaluated using validation datasets to determine their authenticity. Due to narrowed feature utilization, complete understanding of CPPs was not accomplished in some predictors. To overcome these impediments, the development of bioinformatics tools with definite accuracy is essential to overcome the narrowed feature utilization, which favors outstanding CPP identification in the future. Given the significant therapeutic potential of CPPs, particularly in drug delivery, identifying novel and highly efficient CPPs has become a critical requirement. However, this process is exceedingly challenging for biologists. It involves scanning entire proteins using overlapping window patterns and testing each peptide for cell-penetrating activity—a highly labor-intensive and time-consuming endeavor. A computational approach that could predict whether a peptide sequence qualifies as a CPP with good uptake ability would greatly aid biologists by enabling rapid pre-synthesis screening, thereby accelerating CPP-focused research in the future.
